# Clinical Presentation and Management of Breast Cancer With Vertebral Metastasis: A Retrospective Cohort Study in Tunisia

**DOI:** 10.7759/cureus.91113

**Published:** 2025-08-27

**Authors:** Aloulou S Samir, Kammoun Brahim, Bahri Manel, Hamdani Moez, Kraiem Marwa, Harish Rangareddy, Rajhi Hayfa

**Affiliations:** 1 Medical Oncology, Gabes University Hospital, Gabes, TUN; 2 Medical Oncology, University of Sfax, Gabes, TUN; 3 Neurological Surgery, Habib Bourguiba University Hospital, Sfax, TUN; 4 Radiation Oncology, Gabes University Hospital, Gabes, TUN; 5 Pathology, Sadok Mkaddem Hospital, Djerba, TUN; 6 Radiology, Gabes University Hospital, Gabes, TUN; 7 Biochemistry, Haveri Institute of Medical Sciences, Haveri, IND; 8 Medical Analysis, Gabes University Hospital, Gabes, TUN

**Keywords:** bisphosphonates, cancer, chemoterapy, hormonal therapy, metastases, prognosis, radiotherapy, spine, survival

## Abstract

Background

Breast cancer is the most common malignancy affecting women, and vertebral metastases are a frequent complication. This study aimed to examine the clinical and anatomical characteristics, therapeutic management, and prognostic factors associated with vertebral metastases from breast carcinomas in patients from south-eastern Tunisia.

Materials and methods

This descriptive cohort analytical study utilized retrospectively collected data and was conducted in the medical oncology and orthopaedic surgery departments at the Gabes University Hospital over a five-year period, from January 2015 to December 2019. Clinical, pathological, and therapeutic data were statistically analysed to identify prognostic factors influencing overall survival.

Results

The study included 57 patients with a median age of 48 years. Metastases were metachronous in 41 patients (71.9%) and synchronous in 16 (28.1%). Tumours were classified as T4 in 42.1% of study cases. Spinal metastases mainly affected two vertebral levels in 61.4% of patients and involved three vertebrae in 70.2% of cases. Spinal cord compression was the most common complication (24.6%). Extravertebral bone, pulmonary, hepatic, and brain metastases were observed in 80.7%, 43.9%, 49.1%, and 12.3% of patients. Hormone receptors and human epidermal growth factor receptor (HER2) were expressed in 73.3% and 19.3% of cases, respectively. No patients underwent surgery for spinal metastasis. Breast radiotherapy was administered in 52.6% of cases, of which 42.1% targeted spinal metastases. Median survival was 28 months. Overall survival at three and five years was 38.6% and 22.7%, respectively. Significant prognostic factors for overall survival included age (p=0.041) and metastatic disease at diagnosis (p=0.01).

Conclusion

Vertebral bone metastases can compromise the neurological outcomes. However, various systemic and local therapeutic options are available for managing vertebral metastasis. A multidisciplinary approach is essential to optimise strategies and improve prognosis.

## Introduction

Breast cancer is the most commonly diagnosed malignancy and the second leading cause of death among women in the Western world [1]. Spinal metastases occur frequently in breast cancer, accounting for approximately two-thirds of all bone metastases [2]. The incidence of spinal metastases from breast cancer is rising due to the increasing survival of patients with primary breast cancer [2]. Bone metastases can result in skeletal-related events [3], including hypercalcemia, pathological fractures, spinal cord compression, and pain, all of which significantly impair quality of life [4].

The management of spinal metastases requires a multidisciplinary approach that can be broadly categorized into systemic and local treatments. Systemic treatment includes chemotherapy, hormone therapy, targeted therapy, immunotherapy, bisphosphonates, analgesics, and corticosteroids. Local treatment comprises radiotherapy (RT) and surgery [5].

This study aimed to examine the anatomical-clinical characteristics, therapeutic management, and prognostic factors associated with vertebral metastases from breast carcinoma in patients from south-eastern Tunisia.

## Materials and methods

Patient consent and IRC approval

A descriptive cohort analytical study with retrospective data collection was conducted in the medical oncology and orthopaedic surgery departments of Gabes University Hospital between January 2015 and December 2019. The study population comprised hospitalized patients diagnosed with breast cancer, specifically those with vertebral metastatic involvement.

The inclusion criteria consisted of histologically confirmed cases of breast carcinoma, irrespective of patient age, provided there was evidence of vertebral metastases. The presence of vertebral metastases was established through at least one of the following imaging modalities: computed tomography (CT), magnetic resonance imaging (MRI), or bone scintigraphy. All included cases underwent immunohistochemical analysis to determine the expression of estrogen receptors (ER), progesterone receptors (PR), human epidermal growth factor receptor 2 (HER2), and the proliferation marker Ki-67.

Hormone receptor positivity was defined by a PR expression level greater than 1%. HER2 positivity was initially determined by the presence of intense, complete membrane staining in a minimum of 30% of tumor cells. From 2015 onwards, a revised threshold of 10% was implemented for HER2 assessment. In instances where HER2 expression was equivocal, fluorescence in situ hybridization (FISH) was performed to confirm gene amplification. The Ki-67 proliferation index was evaluated according to the laboratory’s internal standards, with a cut-off value of 20% [6].

Patients were excluded from the study if they had bone and/or visceral metastases without spinal involvement, if essential diagnostic data such as spinal imaging, histopathological confirmation, or immunohistochemical results were unavailable, or if the diagnosis pertained to male breast cancer.

Data collection

Clinical data collected included demographics (age, area of residence, socioeconomic status), medical history (comorbidities and menopausal status), and presenting symptoms and signs at hospital admission. Additional data included diagnostic details, location, and number of spinal metastases, associated complications, bone metastases at other skeletal sites, and visceral metastases. Primary breast cancer characteristics were also recorded, including tumour location, size, and staging according to the tumour, node, metastasis (TNM) classification system. Histopathological features included histological subtype, Scarff-Bloom-Richardson (SBR) histoprognostic grade, hormone receptor status, HER2 status, Ki67 value, and molecular subtype.

Statistical analysis

Clinical and pathological variables were assessed to identify factors associated with prognosis. Patient-related variables included age, menopausal status, and the interval between the diagnosis of primary breast cancer and the onset of spinal metastases. Tumour-related variables included tumour size, lymph node involvement, SBR histoprognostic grade, histopathological subtype, vascular emboli, hormone receptor status, extraspinal bone metastases, and visceral metastases.

Data were analysed using SPSS software version 20.0 (IBM Corp, Armonk, NY). Mean, standard deviations, and ranges ​​for quantitative variables with a normal distribution. For non-normally distributed data, medians with minimum and maximum were reported. Qualitative variables were summarized using frequencies and percentages. Overall survival (OS) was the time from the diagnosis of spinal metastasis to the date of last follow-up or death. OS was estimated using the Kaplan-Meier method and assessed based on various prognostic factors. A p-value ≤0.05 was considered statistically significant. For patients who died from causes unrelated to cancer, December 31, 2020 was used as the censoring date.

## Results

Patients and tumour characteristics 

The study included 57 patients who presented to the medical oncology and orthopaedic surgery departments of Gabes University Hospital between January 2015 and December 2019. The median age was 48 years (range: 28−67 years. A majority of patients (57.9 %) were from Gabes, Tunisia. A family history of breast cancer was present in 5.3% of cases, and 61.4% of patients were postmenopausal.

Table 1 presents the tumour characteristics. Based on the 2010 TNM 2010 classification, tumours were staged as T4 in 42.1% of patients, T3 in 22.8%, and T2 in 35.1%. Inflammatory breast cancer (T4d) was observed in 15.8% of the cases.

**Table 1:** Clinicopathological Characteristics of Patients with Vertebral Metastases from Breast Carcinoma (N=57) This table summarizes the clinicopathological characteristics of patients diagnosed with vertebral metastases from breast carcinoma. T, N, M refer to Tumor size, Lymph Node involvement, and Distant Metastasis, respectively, as per the TNM classification system of the American Joint Committee on Cancer (AJCC). SBR: Scarff-Bloom-Richardson grading system; HER2: human epidermal growth factor receptor 2; Luminal A/B: Molecular subtypes based on hormone receptor and HER2 status. Triple negative: Tumors lacking expression of estrogen receptor (ER), progesterone receptor (PR), and HER2.

Breast Cancer Characteristic	N (%)
T Stage (TNM Classification)	
T2	20 (35.1)
T3	13 (22.8)
T4b	13 (22.8)
T4c	2 (3.5)
T4d	9 (15.8)
N Stage (TNM Classification)	
N0	8 (14.0)
N1	32 (56.1)
N2	7 (12.3)
N3	10 (17.5)
M Stage (TNM Classification)	
M0	26 (45.6)
M1	31 (54.4)
Histological Subtype	
Invasive ductal carcinoma	51 (89.5)
Invasive lobular carcinoma	4 (7.0)
Other	2 (3.5)
Grade (SBR Score)	
SBR I	6 (10.7)
SBR II	31 (53.6)
SBR III	20 (35.7)
Hormone Receptor Status	
Positive	42 (73.3)
Negative	15 (26.7)
HER2 Status	
Overexpressed	11 (19.3)
Not overexpressed	46 (80.7)
Molecular Subtype	
Luminal A	14 (24.6)
Luminal B	22 (38.6)
HER2 overexpressed	11 (19.3)
Triple negative	9 (15.8)

Lymph node involvement was classified as N1 in 56.1%, N2 in 12.8%, and N3 in 17.5 % of cases. The most frequent histological subtype was invasive ductal carcinoma (89.5%). The Scarff-Bloom-Richardson (SBR) histoprognostic grade was SBR II in 53.5% and SBR III in 35.7 % of cases. Hormone receptors were positive in 73.3% of patients. HER2 overexpression was identified in 11 patients (19.3%). The Ki-67 proliferation index was assessed in 54 cases (94.7%), with values > 20% in 35 patients (61.4%). Molecular subtypes were classified as follows: luminal A in 14 patients (24.6 %), luminal B in 22 (38.6 %), HER2-positive in 11 (19.3 %), and triple-negative in 9 (15.8 %). Metastatic disease at diagnosis (M1) was observed in 31 patients (54.4%).

Vertebral metastasis characteristics

**Figure 1:**
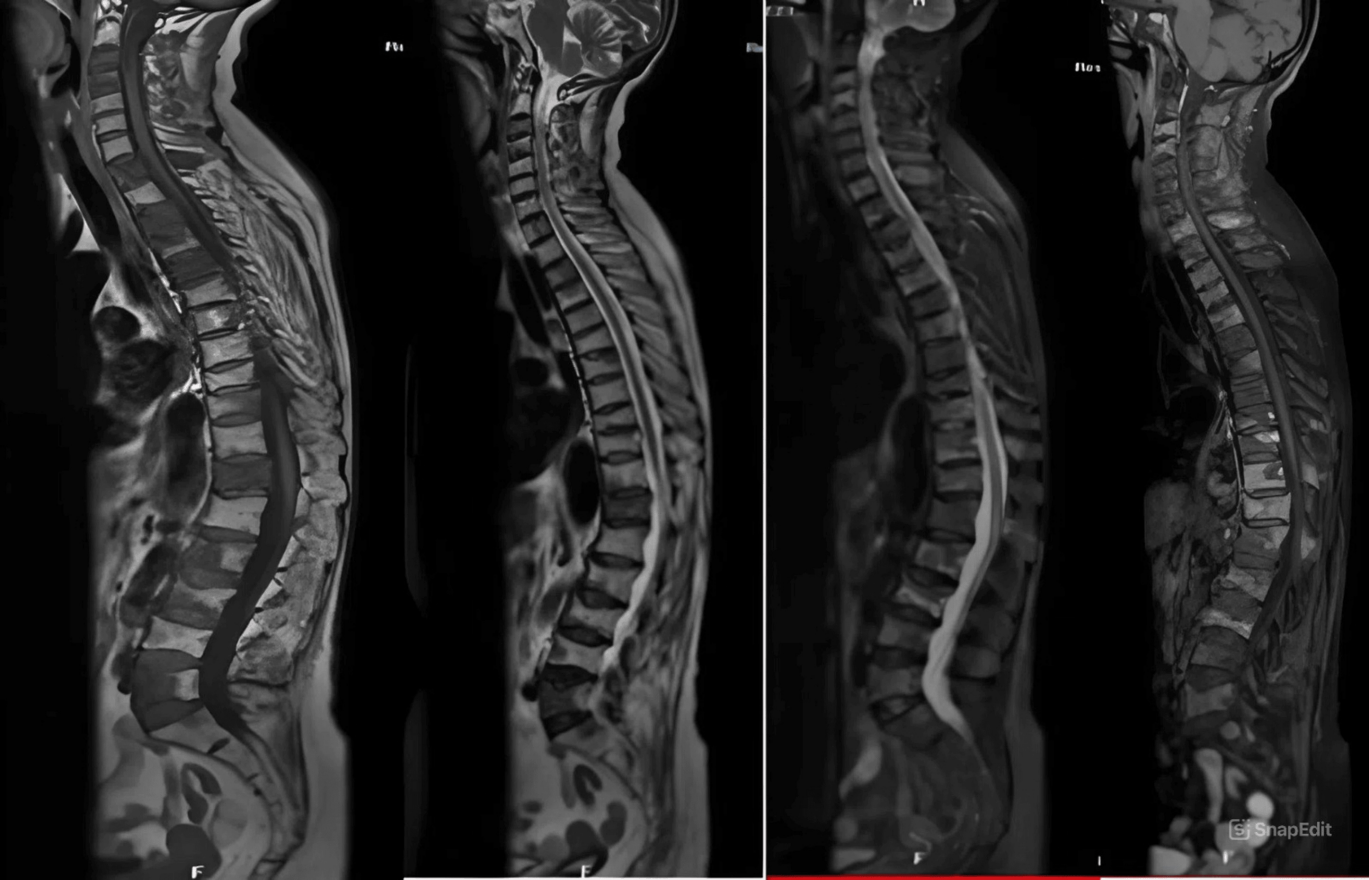
Sagittal MRI images of the spine in a patient with metastatic breast carcinoma. Multiple vertebral bodies across the cervical, thoracic, and lumbar regions exhibit hypointense signals on T1-weighted images and hyperintense signals on short tau inversion recovery (STIR), with intense post-contrast enhancement, indicating widespread vertebral metastases involving both the anterior vertebral bodies and posterior elements. Image: Courtesy of Dr. Samir Aloulou, Department of Medical Oncology, Gabes University Hospital, University of Sfax, Sfax, Tunisia.

The characteristics of vertebral metastasis are shown in Table 2 and Figure 1. Metachronous and synchronous spinal metastases were observed in 71.9% and 28.1% of cases. The average time to diagnosis of spinal metastases was three months. Spinal metastases affected one vertebral level in 12 patients (21.0%), two levels in 35 (61.4%), and three levels in 10 (17.5 %). Regarding vertebral involvement, a single vertebra was affected in nine patients (15.8 %) while more than three vertebrae were involved in 40 patients (70.2%).

**Table 2:** Vertebral Metastasis Characteristics in Patients with Breast Carcinoma (N=57) This table details the vertebral characteristics of metastases observed in patients with breast carcinoma. Skeletal-related events (SREs) include complications such as vertebral fractures, spinal cord compression, and epiduritis (inflammation of the epidural space). Multilevel metastases indicate involvement of more than one vertebral region (e.g., thoracic and lumbosacral). Visceral metastases refer to secondary lesions in organs such as the lungs, liver, or brain.

Vertebral Metastasis Characteristic	N (%)
Number of Spinal Levels Involved	
One	12 (21.1)
Two	35 (61.4)
Three	10 (17.5)
Number of Vertebrae Involved	
One	9 (15.8)
Two or Three	8 (14.0)
More than Three	40 (70.2)
Location of Vertebral Metastases	
Thoracic Spine	6 (10.5)
Lumbosacral Spine	6 (10.5)
Multilevel Metastases	45 (79.0)
Skeletal-Related Events (SREs)	
Vertebral Fracture	5 (8.8)
Spinal Cord Compression	14 (24.6)
Epiduritis	11 (19.3)
Associated Metastases	
Bone (Extraspinal)	46 (80.7)
Visceral (Lung, Liver, Brain)	41 (71.9)

The anatomical distribution included six cases each in the thoracic, lumbar, and sacral spines. Multilevel metastases (either contiguous or non-contiguous) were identified in 45 cases (78.9%). Spinal cord compression was the most frequent complication, diagnosed in 14 patients (24.6%), while epiduritis was observed in 11 patients (19.3%). No cases of hypercalcaemia were reported.

Extravertebral bone metastases occurred in 80.7% of cases, pulmonary metastases in 43.9%, hepatic metastases in 49.1%, and brain metastases in 12.3%.

Treatment and outcomes 

Forty-one patients (71.9%) underwent surgery for primary breast cancer. However, none underwent spinal surgery. Treatment for spinal metastases included systemic therapy: chemotherapy in 45 patients (78.9%) and hormonal therapy in 30 (52.6%). Bisphosphonates were administered in 46 patients (80.7%). Radiotherapy was given in 52.6% of cases; among these, spinal metastases were specifically targeted in 42.1%. Patients underwent three-dimensional (3D) external adjuvant radiotherapy at a dose of 50 Gy in traditional fractionation. Spinal radiotherapy was at a dose of 30 Gy in 10 fractions by two anterior and posterior fields.

At the end of the study period, 44 patients (77.2%) had died due to cancer. The three-year and five-year OS rates were 38.6% and 22.7%, respectively, with a median OS of 44.5 months. Kaplan-Meier method identified two statistically significant prognostic factors associated with OS in univariate analysis: age (p = 0.041) and metastatic disease at diagnosis (p = 0.01) (Table 3).

**Table 3:** Univariate Analysis of Prognostic Factors Affecting Overall Survival (OS) Univariate analysis using the Kaplan-Meier method revealed that OS was significantly influenced by two prognostic factors: Age (p=0.041) and metastatic disease at diagnosis (p=0.01). Other factors such as clinical tumor size, lymph node involvement, histological subtype, menopausal status, histological grade (Scarff-Bloom-Richardson (SBR) grading system), hormone receptor status, extraspinal bone metastasis, associated visceral metastases, and vascular emboli did not show a statistically significant correlation with overall survival (OS).

Variable	p-value	OS Correlation Significance
Age	0.041	Significant
Metastatic disease at diagnosis	0.01	Significant
Clinical tumor size	0.626	Not significant
Lymph node involvement	0.495	Not significant
Histological subtype	0.512	Not significant
Menopausal status	0.107	Not significant
Histological grade (SBR)	0.673	Not significant
Hormone receptor status	0.07	Not significant
Extraspinal bone metastasis	0.296	Not significant
Associated visceral metastases	0.087	Not significant
Vascular emboli	0.83	Not significant

Other factors did not significantly affect OS, including tumour size (p=0.626), lymph node involvement (p=0.495), histological subtype (p=0.512), menopausal status (p=0.107), SBR histoprognostic grade (p=0.673), hormone receptor status (p=0.07), extraspinal bone metastasis (p=0.296), visceral metastases (p=0.087), and vascular emboli (p=0.83). Multivariate analysis did not identify any independent prognostic factors.

## Discussion

This study examined the anatomical-clinical characteristics, therapeutic management, and prognostic factors associated with vertebral metastases from breast carcinomas in patients from south-eastern Tunisia, over five years. It is well established that the most common metastatic sites in breast cancer include the lungs, liver, bones, brain, and lymph nodes, with bone metastases occurring in approximately 70% of cases [3]. Given this high incidence, advancing molecular and genetic diagnosis and therapeutic approaches are essential to improve patient survival [7,8]. Breast cancer is now considered a chronic disease, and the incidence of bone metastasis has been steadily increasing [3]. Bone metastases significantly impair mobility and quality of life in patients with advanced breast cancer [9]. In this study, 71.9% of spinal metastases were metachronous, occurring after initial treatment of the primary tumour. Luminal subtypes were the most common molecular types associated with spinal metastases. Vertebral lesions may result in spinal cord compression, pathological fracture, and instability, leading to severe pain and disability, thereby compromising patients’ quality of life [10-13]. Management of patients with breast cancer metastatic to the bone requires a multidisciplinary approach, with consideration of tumour-specific biology [14].

The treatment of bone metastases is typically palliative, aiming to relieve symptoms, improve quality of life, and potentially prolong survival [9]. Several bone-targeted agents are approved and are currently considered the standard of care [9,15]. The main types include bisphosphonates and the receptor activator of nuclear factor kappa-B ligand (RANKL) inhibitor, denosumab. Bisphosphonates promote osteoclast apoptosis, inhibit bone resorption, and reduce skeletal-related events [14,16,17]. Denosumab, a monoclonal antibody that inhibits osteoclast maturation, has been shown to be more effective than zoledronic acid in preventing skeletal-related events such as pathological fractures, spinal cord compression, and hypercalcemia [9,14,18]. A surgical approach was not employed in the present cohort due to the presence of multifocal spinal metastases and associated visceral involvement.

In this study, the treatment of bone metastasis from breast cancer was based on a combination of chemotherapy, local radiation, hormonal therapy, and regular administration of zoledronic acid. Currently, CDK4/6 inhibitors, such as ribociclib, palbociclib, and abemaciclib, are commonly used in the treatment of luminal metastatic breast cancer, which has demonstrated superior efficacy compared with chemotherapy [19,20]. However, these agents were not available during the study period and were not utilized in our series.

## Conclusions

Vertebral bone metastases can compromise neurological outcomes. However, various systemic and local therapeutic options are available for managing vertebral metastasis. An integrated treatment strategy involving a multidisciplinary team is essential to optimise palliative care, prevent skeletal-related events, and enhance the quality of life as well as overall survival.
